# Efficacy of *Laurus nobilis* L. for Tight Junction Protein Imbalance in Leaky Gut Syndrome

**DOI:** 10.3390/nu16091250

**Published:** 2024-04-23

**Authors:** Yelim Shin, Jiyeon Kim, Youngcheon Song, Sangbum Kim, Hyunseok Kong

**Affiliations:** 1 College of Pharmacy, Sahmyook University, Seoul 01795, Republic of Korea; dpfla5566@naver.com (Y.S.); shelly7285@kosabio.com (J.K.); alexsongsu@syu.ac.kr (Y.S.); sbk@syu.ac.kr (S.K.); 2 KOSA BIO Inc., 272, Namyangju-si 12106, Republic of Korea; 3 PADAM Natural Material Research Institute, Sahmyook University, Seoul 01795, Republic of Korea; 4 College of Animal Resources Science, Seoul 01795, Republic of Korea

**Keywords:** leaky gut, tight junction proteins, imbalance, IL-13/STAT-6 pathway, claudin-2, *Laurus nobilis* L.

## Abstract

*Laurus nobilis* L. (LNL) belongs to the evergreen Lauraceae family. It is native to the Mediterranean and widely distributed in the southern United States, Europe, and the Middle East. LNL is rich in active ingredients of the sesquiterpene lactone series and has been reported to have antioxidant, anti-inflammatory, and anticancer effects. And parthenolide, known as a sesquiterpene lactone-based compound, inhibits the activation of lipopolysaccharide-binding protein (LBP), which is a major trigger for leaky gut syndrome. However, the effectiveness of LNL in improving the state of increased intestinal permeability has not yet been reported. Therefore, we demonstrated the efficacy of LNL, which is known to be rich in parthenolide, in improving intestinal permeability induced by IL-13. We investigated the improvement in permeability and analyzed major tight junction proteins (TJs), permeability-related mechanisms, weight and disease activity indices, and corresponding cytokine mechanisms. LNL maintained TJs homeostasis and clinical improvement by reducing increased claudin-2 through the inhibition of IL-13/STAT6 activation in TJ-damaged conditions. These results are expected to be effective in preventing leaky gut syndrome through the TJ balance and to further improve intestinal-related diseases, such as inflammatory bowel disease.

## 1. Introduction

The intestinal tract is an important organ responsible for digestion, absorption, and excretion of food in daily life. It not only resides in various intestinal microorganisms, including symbiotic bacteria, but also provides an environment in which it is continuously exposed to external pathogens consumed with food. Recently, the importance of intestinal health has emerged [1,2]. If the immune tolerance of intestinal immune control collapses owing to environmental factors, such as bacterial, viral, and parasitic infections or eating habits, stress, and drugs, it can destroy the homeostasis of intestinal epithelial cells, which are known as the primary barrier against external substances [3]. This can cause an imbalance in tight junction (TJ) proteins of the intestinal epithelium, which act as a barrier between the outside and inside of the intestine [4].

TJs exist in the epithelium, endothelium, and myelinated cells and typically consist of structural surface membrane proteins, such as claudins, occludin, junctional adhesion molecules, and zonula occludens (ZO), forming a network that serves as a physical barrier against harmful substances [5]. They also play an important role in immune regulation by constructing pore and leak pathways and maintaining adequate permeability between cells to selectively transport substances required outside and inside [6,7]. Among them, the homeostasis of TJs present in intestinal epithelial cells is known to be maintained by intestinal mucosal mucins and immunoglobulins [8]. In addition to these factors, if intestinal permeability is increased through zonulin activation due to frequent gluten intake, or if lipopolysaccharide (LPS) is excessively present on toll-like receptor 4 (TLR4) or CD14 when LPS-binding proteins (LBP) bind to bactorial LPS in the intestinal tract, TJs may become unbalanced due to the action of the generated pro-inflammatory cytokines, and intestinal permeability may increase [9,10]. If the TJ imbalance continues, barrier function deteriorates and intestinal permeability increases, causing leaky gut syndrome (LGS), which allows harmful substances to easily penetrate the body [11]. The onset of LGS due to an imbalance in TJs can be related not only to direct intestinal inflammatory diseases such as inflammatory bowel disease, but also to various immune diseases such as rheumatoid arthritis, atopy, and type 2 diabetes due to intestinal toxins that invade the intestine [12,13,14]. Therefore, improving the state of increased intestinal permeability, which is the most important factor in preventing and treating these diseases, and maintaining proper barrier function and intestinal permeability through TJ homeostasis are crucial.

Recently, research on lactic acid bacteria and intestinal microbial metabolites has been actively conducted to maintain intestinal health; however, no effective specific material to restore increased intestinal permeability has been developed [15]. In addition, if probiotics are taken while the inner wall of the intestine is not healthy, they penetrate between the intestines and act as a foreign substance, which can cause acute inflammation; therefore, patients with LGS need to consult a specialist before taking probiotics [16,17]. Mesalazine (ME), a 5-aminosalicylic acid (5-ASA) agent used as a representative treatment for inflammatory bowel disease, has been reported to inhibit the synthesis of inflammatory mediators such as prostaglandin E2 and leukotriene B4 and to improve inflammation by inhibiting NF-κB activation, which is known as a regulatory factor for the production of inflammatory cytokines such as IL-1β TNF-α and IL-6 [18,19]. In addition, it is mainly used to induce and maintain the remission of inflammatory bowel disease, which can be caused by increased intestinal permeability, as its effectiveness has been identified to restore the intestinal mucosa [20]. However, a specific efficacy analysis for increased intestinal permeability remains insufficient, and receiving actual ME prescriptions can be difficult because LGS itself is difficult to diagnose, with symptoms improving in daily life and recurring [21]. In addition, studies on ME side effects, such as headache, diarrhea, dizziness, lung disease, liver disease, myocarditis, and pancreatitis have continuously reported that 20%–40% of patients with unspecific side effects fail to maintain long-term remission [22,23,24]. Therefore, to prevent LGS and further improve diseases such as inflammatory bowel disease, interest in natural materials that complement the shortcomings of lactic acid bacteria, have fewer side effects, and are highly effective is increasing. 

*Laurus nobilis* L. (LNL), which belongs to the Lauraceae family *Laurus* L., originates from the Mediterranean coast and has been widely used as a seasoning and spice in the past [25,26]. In Korea, it is cultivated in southern regions, such as Jeollanam-do and Wando [27]. It is a dicotyledonous plant that releases scent when its wavy edges are rubbed. Although undried leaves have a bitter taste, if the leaves are picked and dried well for 1–2 days, the bitter taste disappears, and dried bay leaves are mainly used [28]. LNL contains large amounts of sesquiterpene lactone-based compounds, including parthenolide, reynosin, santamarine, costunolide, and dehydrocoustus lactone [29]. Among these, costunolide is known to be abundant. According to reports, the sesquiterpene lactone compound has been studied as a preventive and therapeutic agent for other diseases, including asthma, atopic dermatitis, and arthritis, due to its excellent antioxidant, antibacterial, and anti-inflammatory effects [30,31], and research reports have indicated that blood sugar and liver enzyme levels were normalized with oral administration of LNL extracts in a streptozotocin-induced diabetes animal model [32]. In addition, parthenolide, which contains a large amount of LNL, has been reported to affect immune regulation while inhibiting the presentation of LPS by being involved in the activating LBP, which causes increased intestinal permeability [33]. However, few studies have evaluated the efficacy of LNL in improving TJ imbalance and intestinal permeability in LGS characterized by Th2-dominant immunity. Therefore, we conducted this study to demonstrate the effectiveness of LNL in improving LGS, which is the leading cause of various intestinal inflammatory diseases, through the improvement of increased intestinal permeability status using Caco-2 cells and oxazolone-induced colitis mouse (OXAC), as well as an analysis of intestinal immunomodulatory mechanisms.

## 2. Materials and Methods

### 2.1. Preparation of Extracts from Laurus nobilis L. (LNL)

The dried LNL leaves were grown in Turkiye and purchased from Bibong Herb (Yangju, Gyeonggi-do, Republic of Korea). They were then extracted using an ethanol solution at 60 C for 3 h. The extract was then freeze-dried and powdered (with yields of 20.2%). All the processes were performed in a good manufacturing practice production facility (Medience Co., Ltd., Chuncheon-si, Republic of Korea).

### 2.2. In Vitro Experiments (Caco-2)

Human Caco-2 cells were purchased from the Korea Cell Line Bank (KCLB, Seoul, Republic of Korea) and cultured in Eagle’s Minimum Essential Medium (EMEM) containing 10% fetal bovine serum (FBS, Hyclone, Rogan, UT, USA) and 1% penicillin–streptomycin (P/S) (GenDEPOT, Barker, TX, USA). All cell culture processes, including subcultures, were maintained at 37 °C in 5% CO_2_ incubator (Heracell 150i, Thermo Fisher Scientific, Waltham, MA, USA) conditions. The culture medium was changed every two days, and after the cells differentiated on the bottom of the T75 flask (Corning, CA, USA) and reached 80–90% confluency, 0.05% Trypsin EDTA (ethylene diamine tetraacetic acid, Corning, CA, USA) was used to harvest the cells.

### 2.3. Cell Viability (MTT Assay)

Cell viability at each concentration of LNL was measured using an MTT assay. Caco-2 cells (1 × 10^4^/well) were seeded in a 96-well plate and cultured until 80–90% confluency was reached. LNL was diluted with an FBS-added medium according to the concentration (12.5, 25, 50, and 100 μg/mL). Subsequently, 200 μL of the diluted LNL was added to each well and incubated for 18 h. After removing the culture medium, 20 μL of a 5 mg/mL MTT [3-(4,5-dimethylthiazol-2-yl)-2,5 diphenyltetrazolium bromide] (Duchefa biochemie, Haarlem, The Netherlands) solution was added and incubated at 37 °C with 5% CO_2_ for 4 h. After removing the MTT solution, 150 μL of dimethyl sulfoxide (DMSO, Sigma, St. Louis, MO, USA) was added and left to react at room temperature for 10 min, and then the optical density was measured at 590 nm using a plate reader (Synergy HTX Multi-Mode Reader, Biotek, CA, USA).

### 2.4. Trans Epithelial Electrical Resistance (TEER) Measurements

For the permeability assays, Caco-2 cells were seeded at 1 × 10^5^ cells/well on Transwell^®^-6well 0.4 μm pore inserts (Corning, NY, USA). The cells were incubated for approximately 21 d to form a monolayer with TJ integrity, and the culture medium was changed every 3 d. To assess whether the cell monolayer was well-formed, whether the TEER value reached 300 Ω·cm^2^ was checked after 21 days using Millicell^®^-ERS (Millipore, Bedford, MA, USA). After the density reached >300 Ω·cm^2^, the culture medium was removed, and LNL (12.5, 25, 50, 100 μg/mL) with 20 ng/mL recombinant human IL-13 (Peprotech, NJ, USA) was added. Thereafter, cells were cultured at 37 °C in a 5% CO_2_ incubator, and the TEER values were measured at 0.5, 1, 2, 4, 6, 12, 24, and 48 h.

TEER value (Ω·cm^2^) = {sample (Ω) − blank (Ω)} × cell growth area (cm^2^)

### 2.5. Real-Time PCR (qPCR)

qRT-PCR was performed to confirm the expression in Caco-2 cells. Cell pretreatment was performed in the same manner as for TEER measurements. Total RNA was isolated using the Total RNA Purification Kit (GeneAll, Seoul, Republic of Korea). For complementary DNA (cDNA) synthesis, 20 μg of RNA was quantified. Afterwards, RNA was mixed with PCR master mix (GeneAll, Seoul, Republic of Korea), oligo(dT) primer and HyperScript™ RT premix (GeneAll, Seoul, Republic of Korea). It was incubated under conditions of 5 min at 42 °C, 60 min at 55 °C, and 5 min at 95 °C. The synthesized cDNA and PCR master mix (AB power STBR^®^ Green, Thermo Fisher Scientific, Waltham, MA, USA) and 0.5 μM of each primer were diluted to perform qRT-PCR (AB step one plus system, Thermo Fisher Scientific, Waltham, MA, USA). Glyceraldehyde-3-phosphate dehydrogenase (GAPDH) was used as an internal control to normalize claudin-1, claudin-2, occludin, and ZO-1 mRNA levels. The results were analyzed using the Quant Studio 5 Real-Time PCR System Software. The primer sequences used for amplification are listed in Table 1.

### 2.6. In Vivo Experiments

**Scheme 1:**
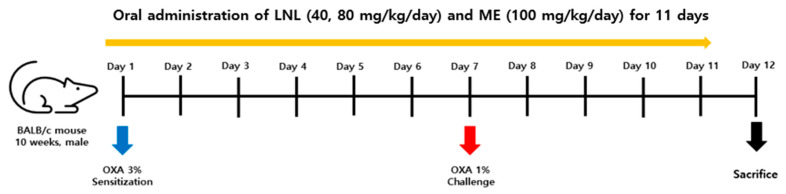
In vivo study scheme.

Male BALB/c mice (9 weeks old, 20–23 g) were purchased from Samtako Co., Ltd. (Osan, Republic of Korea) and acclimatized for 1 week. The mice were kept in individual cages and provided with water and food ad libitum in an SPF animal facility with controlled 12-h photoperiods, and temperature and humidity were controlled at 22 ± 2 °C and 50% ± 20%, respectively. The BALB/c mice were divided into five groups (*n* = 4–6) as follows: normal control (NC), 4-Ethoxymethylene-2-phenyl-2-oxazolin-5-one (OXA, CAS no. 15646-46-5, Sigma, St. Louis, MO, USA)-induced colitis mouse group (OXAC), LNL (40 and 80 mg/kg), and positive control (ME, 100 mg/kg). To increase the body’s sensitivity to OXA, the abdomens of the mice were shaved (2 × 2 cm^2^ area), and 200 μL of a solution containing 3% OXA dissolved in olive oil and acetone (1:4) solvent was applied to the shaved area. After 6 days of OXA sensitization, 100 μL of 1% OXA dissolved in 50% ethanol was administered rectally using a 3 Fr sterilized polyethylene catheter (Lety Medical, Guangzhou, Chjina). During rectal administration, the solution was injected slowly for 30 s, the catheter was removed, and the mice were positioned vertically for 45 s to prevent leakage of the solution through the anus (Scheme 1). Abdominal sensitization and rectal administration were performed in all mice after inducing respiratory anesthesia using isoflurane (3–5%/min, Piramal, Bethlehem, PA, USA). DMSO (2%) was used for all LNL dilutions, and ME was diluted with 1 g of Pentasa^®^ SR granules (Ferring Korea, Seoul, Republic of Korea) in distilled water to yield a 100 mg/kg dose. The respective treatments were orally administered in all five groups once daily for 11 days. The study was conducted in accordance with the ethical guidelines issued by the Institutional Animal Care and Use Committee of Sahmyook University (SYUABR22-004).

### 2.7. Disease Activity Index (DAI)

LNL was administered after OXA abdominal sensitization, and the DAI was recorded after rectal OXA administration until sacrifice. The degree of weight loss, stool patterns, and bleeding were assessed and scored. Weight loss was calculated as a percentage (<1%: 0, 1–5%: 1, 5–10%: 2, 10–20%: 3, and >20%: 4). In addition, the shape of the stool was observed. Soft, viscous, and watery stools and diarrhea were scored as positive (loose stool, 2; diarrhea, 4). It was calculated by classifying when blood in the stool was observed (negative, 0; gross bleeding, 4). The scoring criteria used in the present study have been previously described [34].

### 2.8. IL-5, IL-13, and MPO Enzyme-Linked Immunosorbent Assay (ELISA)

ELISA was performed to determine the cytokine levels and inflammatory activities involved in the induction of colitis. From all the subjects, 50 mg of colon tissues was weighed (AX224KR, OHAUS, Parsippany, NJ, USA) and homogenized in 500 μL of PBS, then frozen at −20 °C (Grand Woosung, Seoul, Republic of Korea) overnight, and the thawing process was repeated twice. To obtain the supernatant, it was centrifuged at 5000 rpm for 5 min at 4 °C. After separation, MPO (R&D Systems, Minneapolis, MN, USA), IL-5, and IL-13 (ELK Biotechnology, Wuhan, China) levels in the supernatant were analyzed using an ELISA kit according to the manufacturer’s protocol provided in the ELISA kit (Melbourne, Australia).

### 2.9. Western Blotting

Western blotting was performed to examine claudin-2 and STAT6 levels in Caco-2 cells and the activation of STAT6 in mouse colon tissues. The cell pretreatment was the same as that used for qRT-PCR. Cells were harvested by adding cell lysis buffer (1×) (GenDEPOT, Katy, TX, USA), Xpert phosphatase inhibitor cocktail solution (100×) (GenDEPOT, TX, USA), and protease inhibitor cocktail solution (100×) (GenDEPOT, Katy, TX, USA). Subsequently, the sample to which the dissolution buffer was added was reacted on ice for 20 min, and then centrifuged at 16,000 rpm at 4 °C for 20 min to obtain a supernatant. The total protein was quantified to 20 μg using the Bradford reagent (GenDEPOT, Katy, TX, USA). Subsequently, a loading sample was prepared using Bolt™ LDS sample buffer 4× (Novex, New Bedford, MA, USA), and sample reduction reagents (Novex, New Bedford, MA, USA) were added and stabilized at 85 °C for 2 min before storing. Mouse colon tissues (100 mg) were weighed and diluted with the same solution used for cell harvesting, then homogenized. After reacting on ice for 2 h, total protein was quantified at 60 μg. The subsequent processes were identical to those used for cell processing.

Electrophoresis was conducted by loading 20 μL of each sample into 4–12% Bolt™ Bis-Trisgel (Invitrogen, Carlsbad, CA, USA). The transfer was performed with iBlot™ 2 PVDF Regular Stack (Invitrogen, Carlsbad, CA, USA), and the blocking process was conducted using 5% bovine serum albumin solution (GenDEPOT, Katy, TX, USA) added to the TBS-T buffer. Primary antibodies including t-STAT6, p-STAT6 (Abcam, Cambridge, UK), claudin-2, and GAPDH (Cellsignaling, Danvers, MA, USA) were then diluted at a ratio of 1:1000 in blocking buffer and incubated overnight at 4 °C. After washing thrice with TBS-T for 5 min, the secondary antibody (Abcam, Cambridge, UK) diluted at a ratio of 1:3000 in blocking buffer was incubated at room temperature for 1 h and washed thrice, and an enhanced chemiluminescence solution (GenDEPOT, Katy, TX, USA) was added. Images were obtained using an imaging system (iBright FL1500, Thermo Fisher Scientific, Waltham, MA, USA).

### 2.10. Immunohistochemistry (IHC) Staining

IHC was performed on mouse colon tissues using claudin-2 (Cell Signaling, E1H9O, 1:1000). Stained slides were scanned using a slide scanner (Carl Zeiss Axio Scan.Z1, Jena, Germany). After scanning, analysis was performed using the Zen Image Analysis software (3.4 blue edition). For the analysis, the positive reaction color of the stained tissue was compared with the total tissue area, and the results are expressed as the total area, positive area, and positive area %.

### 2.11. Statistical Analysis

All results are expressed as mean ± standard error of the mean (SEM), and the significance of differences was analyzed using an unpaired t-test using GraphPad PRISM^®^ Version 5.0 (GraphPad Software; San Diego, CA, USA). Statistical significance was set at *p* < 0.05, *p* < 0.01, and *p* < 0.001.

## 3. Results

### 3.1. In Vitro Experiments

#### 3.1.1. Cell Viability

**Figure 1:**
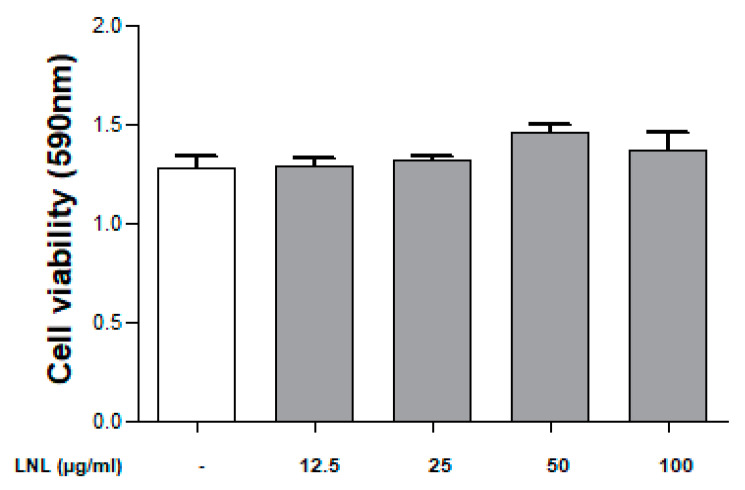
Caco-2 cells were treated with different LNL (concentrations: 12.5, 25, 50, and 100 μg/mL), and cell viability was confirmed using an MTT assay. The cell viability of all groups was compared with the negative. The values are presented as the means ± standard error of the mean.

Toxicity to cells was evaluated according to the concentration of LNL through an MTT assay. When Caco-2 cells were treated with various LNL concentrations (12.5–100 μg/mL) for 18 h, no significant changes were observed at all concentrations (Figure 1).

#### 3.1.2. Efficacy of LNL on TEER in Caco-2 Cells Treated with IL-13

**Figure 2:**
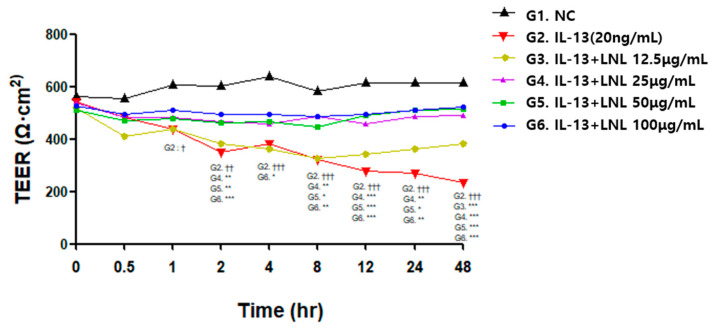
Trans-epithelial electrical resistance (TEER) values were measured for 48 h. Caco-2 cells treated with LNL (12.5, 25, 50, and 100 μg/mL) were treated with IL-13 (20 ng/mL) and cultured in 6-well Transwell plates. Thereafter, the blank TEER value was subtracted from the measured TEER value, and calculations were performed by multiplying the cell growth area. The values are presented as the means ± standard errors of the mean, ^†^ *p* < 0.05, ^††^ *p* < 0.01, ^†††^ *p* < 0.001, compared with the cell-only group * *p* < 0.05, ** *p* < 0.01, *** *p* < 0.001, compared with the IL-13-stimulated group.

TEER analysis was performed to confirm the permeability caused by damage to the TJs formed in the Caco-2 cell monolayer and the permeability improvement effect after treatment with different concentrations of LNL. When comparing the electrical resistance values (Ω·cm^2^) of the induction group treated with IL-13 (20 ng/mL) with the cell-only treatment group, it was confirmed that the electrical resistance value was significantly reduced in the IL-13-stimulated group. Thus, IL-13 increased the permeability of Caco-2 cells, resulting in decreased electrical resistance, indicating TJ damage. In addition, the electrical resistance values of the LNL treatment group treated with IL-13 were compared with those of the IL-13-stimulated group. Hence, it was confirmed that the electrical resistance values in the LNL (12.5–100 µg/mL) group were significantly increased over time compared with those in the IL-13-induced group (Figure 2).

#### 3.1.3. Effect of LNL on TJs Expression Mechanism in Caco-2 Cells

**Figure 3:**
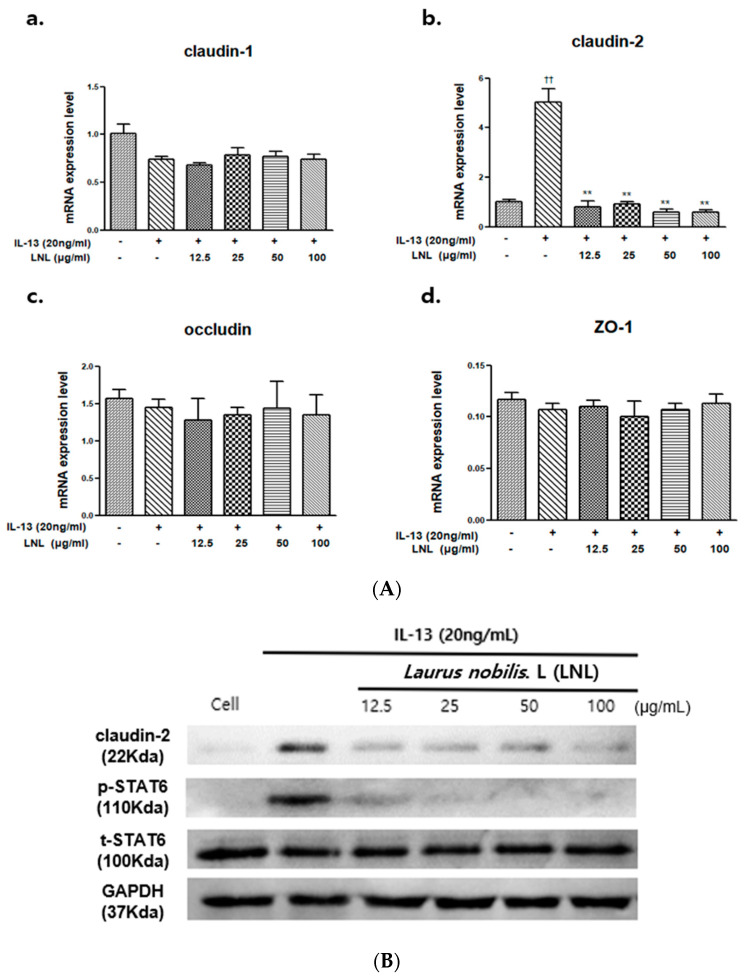
mRNA expression levels in Caco-2 cells (**A**). claudin-1 (**a**), claudin-2 (**b**), occludin (**c**), and ZO-1 (**d**). In all groups, the mRNA of the target gene was analyzed based on glyceraldehyde 3-phosphate dehydrogenase (GAPDH) mRNA expression. The values are presented as means ± standard errors of the mean. ^††^: *p* < 0.01 compared with the cell-only group as the negative control. **: *p* < 0.01 compared with the IL-13-stimulated group. The expression levels of claudin-2 and STAT6 protein were evaluated by Western blotting (**B**).

To analyze the cause of the increased permeability of IL-13-stimulated Caco-2 cells and the improved permeability in the LNL treatment group, the mRNA expression levels of the major TJs (claudin-1, claudin-2, occludin, and ZO-1) were confirmed (Figure 3A). The mRNA levels of claudin-1, occludin, and ZO-1 were not significantly different among the LNL groups. However, when claudin-2 expression was compared between the cell-only and IL-13-stimulated groups, it was confirmed that claudin-2 expression was significantly increased in the IL-13-stimulated group. This indicates that stimulation with IL-13 upregulates the expression of claudin-2. Subsequently, when the IL-13-induced and LNL-treated groups were examined, the expression of claudin-2 was significantly reduced in the LNL-treated group and normalized to that of the cell-only group. The effect of LNL on the expression of STAT6, a transcription factor stimulated by IL-13, and claudin-2 expression were confirmed by Western blotting (Figure 3B). The IL-13-stimulated group demonstrated higher claudin-2 and p-STAT6 expression levels than the cell-only group. Therefore, we confirmed a proportional relationship between the IL-13-stimulated increase in claudin-2 and STAT6 activation. Next, when the expression levels of the LNL group were examined, the claudin-2 and p-STAT6 expression levels were reduced compared to those in the induced group.

### 3.2. In Vivo Experiments

#### 3.2.1. Effects of LNL on Body Weight Change and DAI in the OXAC Model

**Figure 4:**
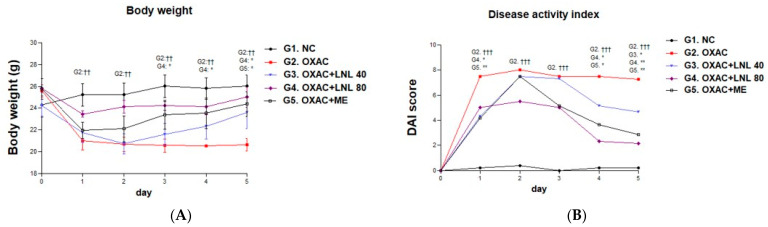
Body weight measurements (**A**) and disease activity index (**B**) were scored once a day after rectal administration of 1% oxazolone-induced colitis. Values are presented as means ± standard errors of the mean. ^††^: *p* < 0.01 and ^†††^: *p* < 0.001 compared with NC; *: *p* < 0.05, **: *p* < 0.01 compared with the OXAC.

From the day after 1% rectal administration, weight loss was observed in all groups except the NC group. In particular, significant weight loss was observed in the OXAC group compared to the NC group. By contrast, the groups administered LNL at different concentrations demonstrated a tendency for body weight to increase. In particular, the LNL 80 group demonstrated a significant increase in weight compared to the OXAC group from the 3rd day to the end of the test, and on the 5th day, the ME group also demonstrated a significant increase compared to the OXAC group (Figure 4A). The DAI tended to increase in all individuals after rectal administration of 1% OXA, and significantly lower DAI scores were observed in the LNL 80 mg and ME groups from day 4 (Figure 4B).

#### 3.2.2. Effects of LNL on IL-5, IL-13, and MPO Secreted from the Large Intestine of OXAC Model

**Figure 5:**
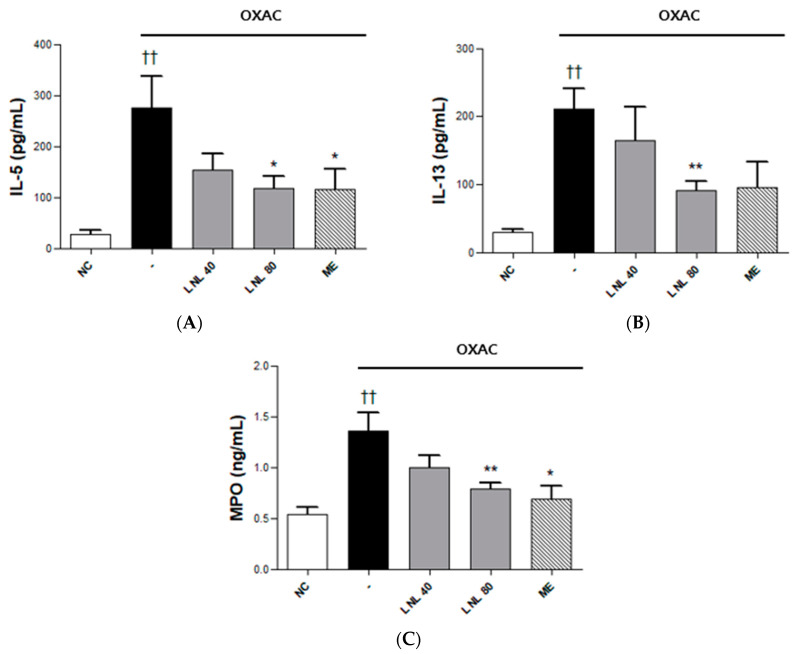
Levels of IL-5 (**A**), IL-13 (**B**), and myeloperoxidase (**C**) in colon tissues from each group were compared using enzyme-linked immunosorbent assay (ELISA). Values are presented as means ± standard errors of the mean. ^††^: *p* < 0.01 compared with the normal control; *: *p* < 0.05, **: *p* < 0.01 compared with OXAC.

The expressions of IL-5, IL-13, and MPO according to the degree of inflammation in the colon tissues of each group were analyzed using ELISA (Figure 5). When comparing IL-5, IL-13, and MPO expression levels between the OXAC and NC groups, all indicators demonstrated a significant tendency to increase. A significant decreasing trend in the IL-5 expression level was observed in the LNL 80 and ME groups compared with the OXAC group (Figure 5A). The IL-13 expression levels were significantly lower in the LNL 80 group compared to the OXAC group (Figure 5B). MPO expression was significantly reduced in the LNL 80 group and ME group than in the other groups (Figure 5C).

#### 3.2.3. Effect of LNL on STAT6 Activation in Mouse Colon Tissue

**Figure 6:**
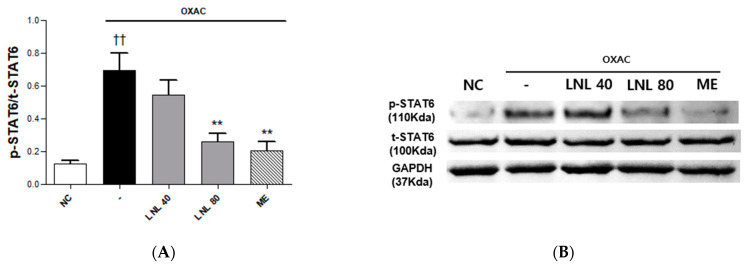
Expression of p-STAT6 in each group was analyzed based on t-STAT6 expression (**A**). Bands representing mean values in each group (**B**); values are presented as means ± standard errors of the mean. ^††^: *p* < 0.01, compared with normal control. **: *p* < 0.01 compared with OXAC.

Based on the results of the cellular experiments, we investigated whether LNL affected STAT6 activation in the colon. The bands in each group were quantified, and the expression level of p-STAT6 was compared with that of t-STAT6. Compared to the NC group, the expression of p-STAT6 was significantly increased in the OXAC group, and a significant decrease was observed in the LNL group. In particular, expression was most suppressed in the LNL 80 mg group, and it was confirmed that it had a similar STAT6 expression inhibitory effect to that in the ME group (Figure 6).

#### 3.2.4. Effect of LNL on Claudin-2 Expression in Mouse Colon Tissue

**Figure 7:**
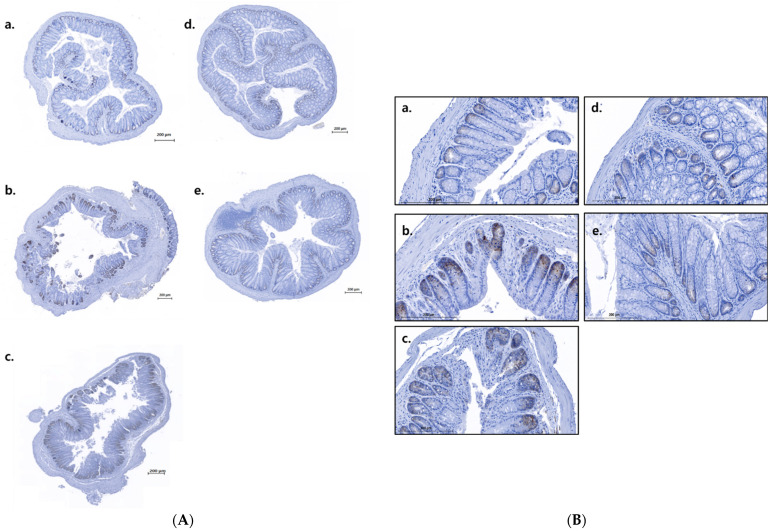
Level of claudin-2 expression in the colon was analyzed using immunohistochemistry (IHC). Colon section (**A**), Magnified section (**B**). NC (**a**), OXAC (**b**), LNL 40 (**c**), LNL 80 (**d**), ME (**e**). Bar = 200 μm. The microscopic images of colon samples obtained using a Carl Zeiss Axio Scan.Z1 are presented.

The expression of claudin-2 in mouse intestinal epithelial cells was analyzed by IHC staining. Claudin-2 expression levels were significantly higher in the OXAC group than in the NC group, whereas they were lower in the LNL 80 and ME groups than in the OXAC group. Claudin-2 expression was confirmed to be similar to that of STAT6 (Table 2 and Figure 7).

## 4. Discussion

A major feature of LGS is an inflammatory response to the invasion of foreign substances due to increased intestinal permeability, which may be attributed to a TJ imbalance [35]. This can lead to various inflammatory diseases, and addressing the imbalance in causative TJs and improving intestinal permeability are important. An imbalance in TJs is caused by the pore and leak pathways of intestinal epithelial cells. The pore pathway is mainly related to expression of the claudin family, whereas the leak pathway is related to occludin, ZO, and myosin light chain kinase [36]. Most of the studies related to intestinal inflammation focus on NF-κB and the AKT/mTOR pathway, which are mainly related to inflammatory cytokines such as TNF-α, IL-1β IL-6, and IL-8, which are Th1-mediated cytokines. This mechanism mainly affects the TJs that constitute the leakage pathway [37,38]. Currently, most drugs used for intestinal inflammatory diseases demonstrate efficacy in the mechanism which is the Th1-mediated cytokine [39]. However, studies on the imbalance of TJs induced by IL-13 in intestinal Th2-dominant immunity, such as ulcerative colitis, are still lacking. Therefore, we determined the efficacy of LNL by focusing on the imbalance in TJs induced by IL-13.

IL-13 is a cytokine secreted by Th2 cells, ILC2, and NKT and is known to be secreted in large quantities in diseases such as asthma and atopic dermatitis [40]. It has also been identified as a major cytokine that affects the intestinal inflammatory response by being oversecreted in ulcerative colitis, where LGS is prominent [41]. Secreted IL-13 binds to the IL-4 receptor alpha (IL-4Ra) and the IL-13Ra-1 co-receptor, or solely to IL-13Ra-2, regulating STAT3 or STAT6 signaling and complementing various biological functions. STAT3 demonstrates heightened sensitivity to various cytokines beyond IL-13, particularly members of the IL-6 family (IL-6, IL-11, IL-31, etc.) and the IL-10 family (IL-10, IL-19, IL-22, and IL-24) [42,43]. Studies on colitis suggest that activation of STAT3 signaling stimulated by IL-22 confers protective effects on the colonic mucosal immune system [44]. In contrast, we prioritized investigating the efficacy of LNL on the IL-13/STAT6 pathway, focusing on pathways that may be more prevalent in Th2-dominant immunity.

Based on the degree of permeability after IL-13 treatment on Caco-2 cells, it was confirmed that the permeability increased over time and the TEER value decreased. Considering the TEER value of the group treated with LNL by concentration, the permeability did not decrease even after IL-13 treatment, and after 48 h, the permeability was significantly improved in all LNL groups compared with that in the IL-13 induction group (Figure 2). To accurately analyze the effectiveness of LNL in improving permeability, major changes in TJs were examined (Figure 3). Among the TJs, claudin-2 belongs to the claudin family and is a protein that makes up the pores between intestinal epithelial cells and plays a role in absorbing ions or moisture from relatively small particles [35]. Several studies have indicated that the expression levels of claudin-2 in the intestine are high in patients with increased intestinal permeability [45], and when treating a claudin-2 knockout mouse model with dextran sulfate sodium to induce colitis, colitis was significantly lower in mice with claudin-2 expression [46]. Therefore, normalizing claudin-2 expression may improve intestinal permeability, thereby alleviating intestinal inflammation. According to our results, the expression of claudin-2 was increased by IL-13, thus confirming a TJ imbalance in Caco-2 cells (Figure 3). By contrast, the group treated with LNL demonstrated a significant decrease in claudin-2 expression, which was increased by IL-13 treatment (Figure 3A(b)). This indicates that LNL may regulate IL-13-induced increases in claudin-2 expression. Also, there were no significant changes observed in the expression of claudin-1, occludin, or ZO-1, excluding claudin-2, which is consistent with previous studies on tight junction proteins in IL-13-induced Caco-2 cells [47,48]. To investigate how LNL affects the regulatory mechanism of claudin-2 expression, we determined the expression levels of STAT6 stimulated by Caco-2 cells in response to IL-13. STAT6 is activated by cytokines such as IL-4 and IL-13 and induces the secretion of Th2-mediated cytokines [49]. It is a member of the STAT family and plays an important role in the initiation of tumor formation and malignant transformation [50], and it has been reported that the rate of progression from ulcerative colitis to colorectal cancer, where the secretion of IL-4 and IL-13 is expressed in a large amount, is higher than that of Crohn’s disease, where Th2 cytokine secretion is relatively small [51]. In addition, when the stimulation of IL-13 induces STAT-6 activation in epithelial cells, it causes an imbalance in claudins, which is an important indicator of intestinal permeability [52]. As demonstrated in Figure 3B, the expression of p-STAT6 in Caco-2 cells treated with IL-13 increased, and the level was normalized in the LNL-treated group. This suggests that IL-13 regulates the expression of claudin-2 through the stimulation of STAT6 and that LNL inhibits the expression of STAT6 and normalizes the expression of claudin-2, helping to balance TJs.

We conducted additional animal experiments using the OXAC model, focusing on investigating claudin-2 expression inhibition and the IL-13/STAT6 signaling pathway, as observed in the results of Caco-2 cell experiments. It induces colitis against the Th2-mediated immune response and is characterized by hypersecretion of IL-13 through the activation of intestinal NKT [53]. Therefore, we used this model to examine the possibility of improving the increased intestinal permeability in an environment similar to that of the IL-13-induced Caco-2 cell efficacy evaluation experiment. It induces colitis against the Th2-mediated immune response and is characterized by the hypersecretion of IL-13 through the activation of intestinal NKT [53]. Therefore, we used this model to examine the possibility of improving the increased intestinal permeability in an environment similar to that of the IL-13-induced Caco-2 cell efficacy evaluation experiment. Furthermore, we used ME, a positive control, as a 5-aminosalicylic acid (5-ASA) drug. ME not only exhibits various anti-inflammatory cytokine inhibition properties, but also regulates the activation of IL-13 receptor alpha-1(IL-13Rα1), as established in previous research [54]. This receptor binds IL-13 and initiates the JAK/STAT signaling pathway. Notably, there is evidence suggesting the involvement of microRNA (miR) 155 regulation in this process. miRNAs, being single-stranded, non-coding RNAs, modulate biological functions by either suppressing translation or promoting the degradation of target mRNAs [19]. Given the close association between miR155 and IL-13 signaling, it is hypothesized that 5-ASA may attenuate miR155 expression, thereby impacting IL-13 receptor expression and activation, consequently affecting JAK/STAT signaling. Therefore, we compared and analyzed the efficacy of LNL on IL-13 signaling with that of ME.

LNL and ME were administered orally after abdominal sensitization for a total of 11 days. Body weight and DAI were compared for 5 days immediately after rectal administration caused colitis. In the OXAC group, significant weight loss and high DAI scores were observed from the first day of workplace induction compared to the NC group, whereas in the LNL group, weight loss improvement was confirmed from the third day after workplace induction. In the LNL 80 group, the DAI was significantly lower than that in the OXAC group from the fourth day. In addition, a significant improvement was observed on day 5 in the ME group (Figure 4). Considering that oral administration was performed from the day of abdominal sensitization to OXA, the LNL 80 group had a rapid recovery rate, suggesting that intestinal inflammation can be prevented and improved through effective intestinal immune control before and after colitis induction.

An examination of the expression levels of inflammatory cytokines involved in intestinal inflammation in OXAC confirmed that colitis, which demonstrates a pattern of hypersecretion of IL-5 and IL-13, was induced (Figure 5). IL-5 is known to induce Th2-mediated cytokine secretion and the recruitment and activation of eosinophils through Th2 stimulation, stimulation of mast cells in the colonic mucosa, and catalysis of the intestinal inflammatory response through histamine secretion and activation of prostaglandin E2 and leukotriene [55]. When the expression level of IL-5 was evaluated (Figure 5A), a significant increase was demonstrated in the OXAC group, a concentration-dependent decrease was confirmed in the LNL group, and, in particular, it was significantly reduced in the LN 80 and ME groups. This indicates that LNL can inhibit IL-5-induced eosinophil activation and mast cell stimulation as well as the release of inflammatory mediators. IL-13 is known to have a great effect on intestinal permeability by activating the caspase-3 mechanism through the tumor necrosis factor-like indicator of apoptosis/fibroblast growth factor-inducible 14 pathway, causing the death of intestinal epithelial cells. In this experiment [56], it was confirmed through Caco-2 cells induced by IL-13 that the activation of STAT6 causes overexpression of claudin-2 to induce increased intestinal permeability. When the expression level of IL-13 was evaluated (Figure 5B), a significant increase was observed in the OXAC group, and the LNL administration group tended to decrease in a concentration-dependent manner; in particular, it was significantly reduced in the LNL 80 group. This suggests that the administration of 80 mg LNL inhibited the secretion of IL-13, thereby ameliorating the increase in intestinal permeability through the regulation of claudin-2 expression. MPO is an enzyme produced by neutrophils and secreted during NETosis, which is a major indicator of neutrophil infiltration and accumulation [57]. It has been reported that neutrophils secrete various inflammatory cytokines when activated as immune cells that initiate and continue the initial inflammatory response to external substances in the intestine, and help to activate other immune cells to induce an inflammatory response [58]. The expression level of MPO was significantly increased in the OXAC group (Figure 5C) and was significantly lower in the LNL 80 and ME groups. These results indicate that LNL can inhibit the inflammatory response to neutrophils, suggesting that it exhibits efficacy similar to that of ME in improving overall intestinal inflammation.

Considering the cellular results indicating that the secretion of IL-13 influences claudin-2 regulation through STAT6 stimulation, the expression of STAT6 in mouse colon tissues was compared (Figure 6). The expression of P-STAT6 was significantly lower in the LNL 80 group, which confirmed a significant reduction in IL-13 levels (Figure 6A). Hence, the LNL 80 group demonstrated a significant reduction in STAT6 through the inhibitory ability of IL-13, suggesting that the overexpression of claudin-2 among the causes of TJ imbalance in intestinal epithelial cells can be controlled, and furthermore, it implies the possibility of inhibiting tumor production and maintenance through IL-13/STAT6 pathway overactivation. Further research is needed. Subsequently, when the expression of claudin-2 was confirmed by IHC analysis of the colon tissue of each group (Figure 7), the OXAC group demonstrated the highest expression level in the positive area of claudin-2 compared to the total area, and the expression of claudin-2 in the LNL 80 group, which demonstrated a significant reduction in p-STAT6, was similar to that in the NC group (Table 2). This demonstrates that LNL can improve intestinal permeability by regulating the expression of claudin-2 through the inhibition of STAT6 activation in the body.

Polyphenols and sesquiterpene lactones, known to be abundant in LNL, have been shown to possess inhibitory effects on the JAK/STAT pathway [59]. Among them, costunolide has been found to influence CD4+ T cell regulation, affecting T cell differentiation and thereby demonstrating efficacy in reducing IL-13 secretion [60]. Additionally, research has indicated that a significant decrease in IL-13 levels occurred with a reduction the secretion of eosinophils, which produce large amounts of IL-4 and IL-13, in an OVA-induced asthma mouse model [61]. These findings underscore the need for further comparative analysis between LNL and individual constituents such as polyphenols and sesquiterpene lactones to elucidate the specific mechanisms and efficacy of LNL in improving intestinal permeability. Moreover, considering the observed similarity in efficacy between LNL and ME, investigating the efficacy of IL-13Ra1 and IL-13Ra2, with reference to the mechanism by which ME regulates the IL-13/STAT6 pathway, may provide a basis for establishing the mechanism of action of LNL on the IL-13/STAT6 pathway.

In this study, we confirmed that LNL can improve the imbalance of TJs to IL-13 in Caco-2 cells and demonstrated efficacy in clinical improvement and intestinal inflammation relief through the inhibition of claudin-2 expression in actual OXAC bodies. Overall, LNL plays an important role in normalizing the expression of claudin-2 through the inhibition of the IL-13/STAT-6 pathway, demonstrating the possibility of improving intestinal permeability by balancing TJs. These results suggest the possibility of using LNL as a new intestinal-related functional food as an alternative to lactic acid bacteria, a representative intestinal health-related functional food. Specific studies are also needed to help create an environment in which lactobacilli can fully settle and multiply in the intestine by supplementing the shortcomings of lactobacilli with a mixture of LNL and lactic acid bacteria. Furthermore, it has the potential to prevent autoimmune diseases such as inflammatory bowel disease, rheumatoid arthritis, atopy, and allergies, which can be caused by increased intestinal permeability, suggesting the possibility of developing it as a new treatment for intestinal inflammation caused by inflammation-mediated cytokines.

## 5. Conclusions

We aimed to validate the novel efficacy of LNL in a model representing intestinal TJ imbalance. Using Caco-2 cells and the OXAC model, we confirmed the efficacy of LNL in inhibiting Claudin-2 expression through regulation of the IL-13/STAT6 pathway. Particularly, administration of LNL orally in the OXAC model demonstrated improvements in weight loss and DAI, and we proved the ability to suppress intestinal Claudin-2 expression through IL-13/STAT6 regulation. Our study highlights the potential of LNL as a therapeutic paradigm for conditions characterized by TJ imbalance.

## Figures and Tables

**Table 1:** Primers used for real-time PCR analysis of gene expression.

Target Gene	Primer Sequence 5′ > 3′	
	Forward	Reverse
claudin-1	CCTCCTGGGAGTGATAGCAAT	GGCAACTAAAATAGCCAGACCT
claudin-2	GCCTCTGGATGGAATGTGCC	GCTCCGCCACTCTGTCTTTG
occludin	GACTTCAGGCAGCCTCGTTAC	GCCAGTTGTGTAGTCGTGCTCA
ZO-1	ACCAGTAAGTCGTCCTGATCC	TCGGCCAAATCTTCTCACTCC
GAPDH	CAACTTTGGCATTGTGGAAG	GGAGGAAGAGGATGCGGCAGT

**Table 2:** Effect of claudin-2 expression on the colon.

	NC	OXAC	LNL 40	LNL 80	ME
**Total** **a** **rea (μm^2^)**	1,433,933.28	3,065,461.29	2,546,376.58	2,375,414.46	2,208,251.85
**claudin-2** **a** **rea (μm^2^)**	24,089.25	95,752.78	66,821.68	35,863.96	33,907.18
**claudin-2** **a** **rea (%)**	1.68	3.12	2.62	1.51	1.54

## Data Availability

The datasets used and/or analyzed during the current study are available from the corresponding author upon reasonable request.
